# Determinants of Left Ventricular Dysfunction and Remodeling in Patients With Corrected Tetralogy of Fallot

**DOI:** 10.1161/JAHA.118.009618

**Published:** 2019-08-31

**Authors:** Ana Cristina Andrade, Michael Jerosch‐Herold, Philip Wegner, Dominik Daniel Gabbert, Inga Voges, Minh Pham, Ravi Shah, Jürgen Hedderich, Hans‐Heiner Kramer, Carsten Rickers

**Affiliations:** ^1^ Heart Institute Medical School of São Paulo University São Paulo Brazil; ^2^ Department of Congenital Heart Disease and Pediatric Cardiology University Hospital of Schleswig‐Holstein Kiel Germany; ^3^ Department for Medical Informatics and Statistics University Hospital of Schleswig‐Holstein Kiel Germany; ^4^ Department of Radiology Brigham & Women's Hospital and Harvard Medical School Boston MA; ^5^ University Heart Center Adult with Congenital Heart Disease Unit University Hospital Hamburg‐Eppendorf Hamburg Germany

**Keywords:** cardiac magnetic resonance imaging, cardiopulmonary exercise test, heart failure, left ventricular remodeling, tetralogy of Fallot, Congenital Heart Disease

## Abstract

**Background:**

The aim of this study was to identify in asymptomatic patients with repaired tetralogy of Fallot the prevalence and determinants of impaired left‐sided cardiac function and adverse ventricular remodeling and the relation of left ventricular (LV) dysfunction and remodeling with cardiopulmonary exercise capacity.

**Methods and Results:**

In a cross‐sectional study, 103 patients with tetralogy of Fallot (median age, 16.3 years) in New York Heart Association class 1, with surgical repair at a median age of 1.1 years, and 63 age‐matched controls were studied. LV, right ventricular function and geometry, LV myocardial extracellular volume (n=57), and left atrial function were quantified with cardiac magnetic resonance. Peak oxygen consumption was measured by a standardized cardiopulmonary exercise test (n=70). Patients with tetralogy of Fallot had lower LV ejection fraction (*P*=0.001; 49% below age‐adjusted fifth percentile for controls), lower LV mass index (*P*=0.003), lower LV mass/volume ratio (*P*<0.01), and impaired left atrial function. Right ventricular mass/volume ratio was the best predictor for LV systolic dysfunction and for a lower LV mass/volume ratio. Compared with controls, LV extracellular volume was higher (*P*<0.001), particularly in female patients, and associated with subnormal peak oxygen consumption (*P*=0.037). A peak oxygen consumption below the third percentile reference level was more likely with decreasing LV ejection fraction (*P*=0.008), and lower LV mass index (*P*=0.024), but independent of right ventricular ejection fraction.

**Conclusions:**

In New York Heart Association class 1 patients with tetralogy of Fallot, frequent impaired systolic and diastolic LV function, LV adverse remodeling with LV atrophy, a decreased mass/volume ratio, and extracellular matrix expansion suggest cardiomyopathic changes. The best predictor for LV systolic dysfunction was the right ventricular mass/volume ratio. The subnormal peak oxygen consumption indicates that monitoring of LV status may be important for long‐term prognosis.

**Clinical Perspective**

**What Is New?**

- Impaired systolic and diastolic left ventricular (LV) function, adverse LV remodeling with extracellular matrix expansion, reduced cardiomyocyte mass (atrophy), and a decreased mass/volume ratio have been reported in young to middle‐aged patients with tetralogy of Fallot at a median of 14 years after repair, suggesting LV cardiomyopathic changes, despite the absence of any limitations to normal physical activity (New York Heart Association class 1).
- Markers of LV systolic dysfunction and adverse tissue remodeling (reduced cardiomyocyte mass), measured by cardiac magnetic resonance imaging, were associated with subnormal peak oxygen consumption during cardiopulmonary exercise testing, an important risk predictor in tetralogy of Fallot.

**What Are the Clinical Implications?**

- This study recommends imaging‐based surveillance of the LV in patients with repaired tetralogy of Fallot to monitor adverse remodeling.
- Timely initiation of surgical reintervention for pulmonary insufficiency if present, or medical heart failure therapy, may delay progression of adverse remodeling and improve the long‐term prognosis.

## Introduction

In tetralogy of Fallot (ToF), the main clinical focus after surgical repair is directed to the right ventricle (RV) (eg, to the degree of pulmonary regurgitation and RV dilatation).^1^ Left ventricular (LV) dysfunction and remodeling in patients with RV overload has received much less attention, although some studies indicate that it may in the long‐term significantly contribute to left heart failure.^2^, ^3^

Indeed, a recent study identified LV systolic dysfunction as an outcome predictor for death and ventricular tachycardia in ToF.^4^ However, the interrelationship between RV and LV remodeling has not been studied in detail in clinically asymptomatic patients with ToF.

Cardiac magnetic resonance (CMR) is optimally suited to simultaneously assess adverse RV and LV volumes and function.^5^ Emerging CMR techniques allow tissue phenotyping within the heart, including the assessment of diffuse interstitial myocardial fibrosis, by measuring the expansion of the extracellular volume (ECV).^6^

The goals of the present study were as follows: (1) to determine the prevalence of LV dysfunction in NHYA class 1 patients with ToF and to identify associations between right heart markers of pathologic and LV remodeling; and (2) to determine, in a subset of patients (n=70) with cardiopulmonary exercise testing, if LV dysfunction and remodeling have an impact on age‐ and sex‐adjusted peak exercise capacity, a potentially important risk predictor for adverse events in ToF.^7^

## Methods

The data that support the findings of this study are available from the corresponding author on reasonable request.

### Patients and Controls

The cohort for this prospective cross‐sectional study consisted of consecutive patients with ToF without overt symptoms, who underwent CMR imaging between January 2012 and August 2016. Patients with clinical symptoms of New York Heart Association functional class >1 as well as patients taking cardiac medications, like angiotensin‐converting enzyme inhibitors and β blockers, were excluded. The Institutional Review Board of our hospital approved the study (protocol No. A168/07), and informed written consent was obtained from either the patients or their guardians. Age‐matched healthy controls were recruited for comparison with the ToF cohort among patients scheduled to undergo a clinically indicated, contrast‐enhanced neurological magnetic resonance imaging or an magnetic resonance imaging study to rule out a vascular abnormality within the thorax. All individuals in the control group had a normal CMR examination result and no history of left‐sided heart disease or cardiomyopathy.

### Cardiac Magnetic Resonance

All CMR studies were performed with a 1.5‐T scanner (Achieva; Philips Medical Systems, the Netherlands) using a phased‐array coil for cardiac imaging. In patients aged <7 years, sedation with propofol and midazolam was necessary. Heart rate, oxygen saturation, and noninvasive blood pressure were assessed with a CMR‐compatible monitor (Invivo Precess 3160; Invivo, Orlando, FL). Dedicated CMR software (ViewForum release 6.3 [Philips Medical Systems, the Netherlands]; Mass CMR software [Medis, Leiden, the Netherlands) was used to analyze the images.

### LV Function and Volumes

Short‐axis cine imaging was performed to measure LV and RV volumes at end systole (ES) and end diastole (ED), mass at ED, and ejection fraction (EF), per standard guidelines.^8^ Volumes and masses were all indexed to body surface area (BSA). Indexed quantities are denoted by an “i” at the end (eg, LV EDVi for LV ED volume index). Global ventricular remodeling was assessed by the ratio of myocardial mass (LV or RV), divided by the EDV of the LV or RV, respectively. Eccentric remodeling is characterized by a mass/volume ratio that is smaller than in normal controls.^9^, ^10^ Cine imaging was performed with steady‐state free precession (field of view, 280×224 mm; voxel size, 1.88×1.94×6 mm; repetition time/echo time=4.4/2.5 ms; 25 cardiac phases; no breath hold; averages=2; total scan duration, 3–6 minutes) with 8‐mm interslice distance and full LV and RV coverage. Axial cine acquisitions were used to measure left atrial (LA) volumes. The LA volumes were also indexed to BSA and are reported for 3 time points during the cardiac cycle: at maximal LA volume before mitral valve opening (LA_max_), minimal LA volume at mitral valve closure (LA_min_), and LA volume immediately before atrial contraction (LA_bac_). LA volumes and LA functional parameters (including passive, active, and total LA EF) were calculated as described previously.^11^ Total LA emptying volume was defined as the difference between LA_max_ and LA_min_, and was divided into LA passive emptying volume and LA contractile volume. LA contractile volume was the difference between LA_bac_ and LA_min_, and LA passive emptying volume was the difference between LA_max_ and LA_bac_. LA functional parameters were calculated according to the following formulas:

$$\begin{array}{cc} & \text{LA ejection fraction (LA EF)}\;=\;\frac{({\text{LA}}_{\max}-{\text{LA}}_{\min})\times 100\%}{{\text{LA}}_{\max}}\\ & \text{LA passive emptying function (LA PEF)}\\ & \;\;\;\;\;\;=\;\frac{({\text{LA}}_{\max}-{\text{LA}}_{\mathrm{bac}})\times 100\%}{{\text{LA}}_{\max}}\\ & \text{LA contractile emptying function (LA CEF)}\\ & \;\;\;\;\;\;=\;\frac{({\text{LA}}_{\mathrm{bac}}-{\text{LA}}_{\min})\times 100\%}{{\text{LA}}_{\mathrm{bac}}} \end{array}$$

### Phase Contrast Cine Imaging

A retrospectively gated, phase‐contrast cine pulse sequence, with through‐plane velocity encoding, was applied to measure flow (field of view, 270×270 mm; voxel size, 1.64×1.4× 7 mm; repetition time/echo time, 4.4/2.7 ms; velocity encoding 200 cm/s) in the main pulmonary artery, with the slice positioned ≈1.5 cm above the pulmonary valve. Pulmonary regurgitation was calculated as the fraction of forward flow that returned to the ventricle in diastole.^1^

### Late Gadolinium Enhancement

Late gadolinium enhancement (LGE) imaging was performed using the contrast agent gadopentetate dimeglumine (Magnevist; Bayer Schering Pharma AG, Germany), with a contrast bolus of 0.1 mmol/kg. Approximately 15 minutes after contrast administration, LGE images were acquired with an inversion‐recovery–prepared 3‐dimensional gradient echo sequence (field of view, 300× 178×80 mm; voxel size, 1.17×1.27×10 mm; repetition time/echo time, 3.7/1.83 ms; flip angle, 15°; time after inversion (TI) adjusted to null normal myocardium) with respiratory navigator technique. The presence and location of LGE was identified qualitatively by signal enhancement within the myocardium. Some patients or their guardians either declined to receive contrast or woke up from sedation and did not receive contrast agent (36%). Heart‐healthy control subjects did not receive any contrast agent, except for 12 subjects who were scanned for a neurological indication.

### ECV Fraction

Cardiac T1 measurements were performed using a cine Look‐Locker technique^6^, ^12^ for one short‐axis slice in the mid‐LV. This technique uses a non–slice‐selective inversion pulse after the R wave, followed by segmented gradient echo cine acquisition for 20 cardiac phases over 2 to 3 cardiac cycles, representing 20 TI. Parameters are as follows: TI increment, 40 milliseconds; slice thickness, 8 mm; repetition time >3 RR intervals. T1 measurements were performed once before contrast, and at least once after contrast, with the postcontrast acquisition done at least 10 minutes after contrast administration. Look‐Locker images were segmented along the endocardial and epicardial borders of the LV, which was further divided into 6 standard segments. The mean signal intensity in each segment was plotted against TI, and T1 determined with a nonlinear least‐squares algorithm, and correction for radiofrequency pulse effects. The relaxation rate R1 (=1/T1) in the myocardium was plotted against the R1 in the blood pool. The partition coefficient was calculated as the slope of the least‐squares regression line when the number of T1 measurements was >2, or by calculating the ratio of the precontrast/postcontrast R1 change in myocardium, and the R1 change in blood, when only one precontrast and one postcontrast measurement were made. The ECV (for each segment) was calculated as the product of the partition coefficient and (1‐hematocrit), as previously described.^6^, ^13^, ^14^ We used a global ECV for the mid‐LV slice, calculated as the average of the segmental ECV values.

### Cardiopulmonary Exercise Testing

Peak oxygen consumption (VO_2_ max) was measured by a standard exercise test^15^, ^16^ within 6 months before or after the date of magnetic resonance imaging. VO_2_ max was obtained in mL kg^−1^ min^−1^, and expressed as age‐ and sex‐adjusted percentile ranking on the basis of previously published cardiopulmonary exercise testing (CPET) reference data from 548 female and 647 male patients within 4 to 75 years of age.^15^ Subnormal exercise capacity was defined as VO_2_ max lower than the third percentile levels for a normal reference population.^15^, ^17^

### Statistical Analysis

Statistical analysis was performed using MedCalc, version 11.5.1.0 (Mariakerke, Belgium), and the R language and environment for statistical computing (R, version 3.4.3; R Foundation for Statistical Computing, Vienna, Austria; 2017).^18^ Data are reported as mean±SD. The Wilcoxon‐Mann‐Whitney test, or Fisher's exact test for categorical variables, was used to compare data for patients and healthy subjects. The strength of correlations between variables was assessed with Spearman's rank correlation. All comparison tests were 2 tailed, and *P*<0.05 was considered statistically significant. Jonckheere nonparametric test was used to check for trend of increasing or decreasing values across *ordered* levels of systolic function, ranging from normal, through mild to moderate dysfunction.

As most LV parameters in normal controls show a significant association with age, we defined their normal ranges as a function of age. Smooth curves, corresponding to the 5th and 95th percentile levels for LV parameters measured in healthy volunteers, were calculated with generalized additive models for location scale and shape, using a spline for age as single predictor (package “gamlss” in R). As all controls who had ECV measurements in our study were aged <18 years, we used published referenced data^19^ for ECV in adults, and estimated the normal range from means and SDs, to determine the proportion of patients with ToF and ECV above the normal range in their age group (<18, 18–40, 40–60, or >60 years).

Multivariate regression models were built for LV EF and LV mass/volume ratio, to determine which measures of right‐sided dysfunction were associated with LV dysfunction and LV remodeling. Age, sex, BSA‐indexed RV volumes at ED and ES, RV mass, RV mass/volume ratio, LV/RV mass ratio, and RV peak pressure from a contemporaneous Doppler‐echocardiography examination were considered as predictors. A best model was chosen by step‐wise forward and backward predictor selection, using the Akaike information criterion. Associations between the prevalence of eccentric LV remodeling and continuous RV parameters were analyzed by logistic regression analysis. A multivariate model for VO_2_ max (in mL/kg per minute) included age, sex, LV EF, and cardiomyocyte mass index as predictors (ie, the univariate predictors, other than age and sex, that had a significant effect on abnormal exercise capacity by univariate analysis). (LV mass was not included as simultaneous predictor because of a relatively strong collinearity with cardiomyocyte mass index.) Preliminary analysis suggested that some of the relationships with VO_2_ max appeared to be nonlinear. Therefore, we used a generalized additive model and represented age, LV EF, and cardiomyocyte mass index by cubic regression splines to account for these nonlinearities. Multivariate logistic regression analysis was used to identify predictors of abnormally low VO_2_ max.

## Results

### Baseline Characteristics

A total of 103 patients with ToF (43 female patients, 60 male patients; all New York Heart Association class 1) were studied at 15.1±8.7 years (median, 14.3 years; range, 1.3–39 years) after surgical repair of ToF. The patient characteristics are summarized in Table 1 and compared with 63 healthy controls. Twenty patients initially had a shunt implantation (17 Blalock‐Taussig shunts and 3 Waterston shunts). In 51 patients (49%), a transannular patch was used for RV outflow tract reconstruction. A total of 25 patients (24%) underwent a reoperation of pulmonary insufficiency. Of those patients, 14 (56%) received a valved RV pulmonary artery conduit and 11 (44%) underwent a reconstruction of the pulmonary valve. Twelve patients (11%) had a QRS duration >180 milliseconds. There were no differences in age, weight, height, BSA, and blood pressure between patients and healthy subjects (Table 1). CPET was performed in 70 patients (68%). A total of 20 (29%) of 70 patients with ToF had an abnormally low VO_2_ max value.

**Table 1:** Clinical Characteristics of Patients With ToF and Controls

Variable	Patients With ToF (n=103)	Controls (n=63)	*P* Value
Age, y	16.27 (10.86)	16.15 (17.94)	0.46
Female sex	43 (42)	36 (57)	0.08
Age at repair, y	1.04 (1.65)	…	…
Weight, kg	52.8±24.6	51.9±22.5	0.82
Height, cm	153±29.5	157±26.6	0.384
BSA, m^2^	1.47±0.49	1.49±0.45	0.986
Heart rate, bpm	77.4±13.5	74.2±17	0.115
SBP, mm Hg	107±18.6	105±10.2	0.144
DBP, mm Hg	57.8±12	60.4±11.7	0.289
TAP	51 (49)	…	…
Reoperation	25 (24)	…	…
Valved RV PA grafts	14 (13.6)	…	…
PV reconstruction	11 (10.7)	…	…
Maximum RVp, mm Hg	26.5±8.1	…	…

Data are presented as mean±SD, median (interquartile range) for age, or number (percentage). *P* values are from the Wilcoxon test and Fisher exact test (sex). Bpm indicates beats per minute; BSA, body surface area; DBP, diastolic blood pressure; PA, pulmonary artery; PV, pulmonary valve; RV, right ventricle; RVp, RV pressure (estimated from tricuspid insufficiency by Doppler echocardiography); SBP, systolic blood pressure; TAP, transannular patch during first operation; ToF, tetralogy of Fallot.

### Ventricular Volumes and Function

**Figure 1:**
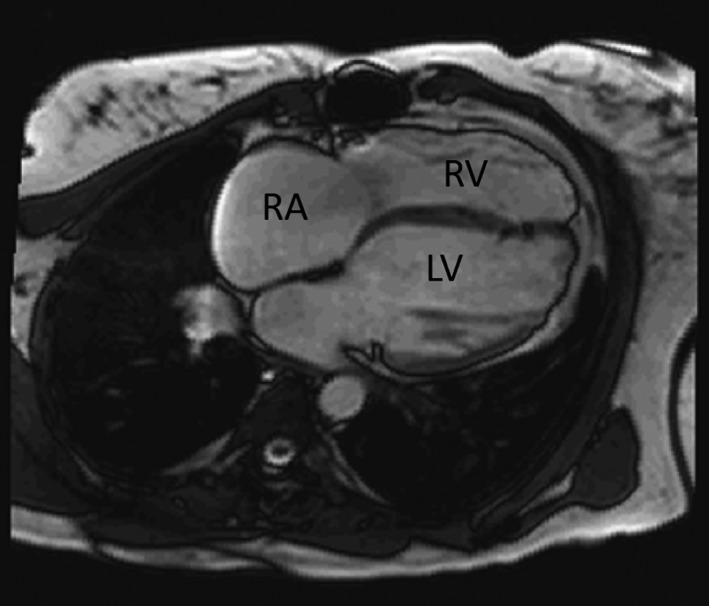
The 4‐chamber view from cine magnetic resonance imaging of a 26‐year‐old asymptomatic female patient with tetralogy of Fallot illustrates dilated left ventricle (LV), right ventricle (RV), and right atrium (RA) and a thinned LV wall, without evidence of any scar on late‐gadolinium imaging. Systolic LV function was borderline (LV ejection fraction, 50%), and the LV mass index (40 g/m²) was below the normal reference range^20^ (median, 62 g/m²; 95% CI, 47–77 g/m²).

Figure 1 shows a 4‐chamber end‐diastolic view from a cine acquisition, with dilated right and LVs, borderline LV EF, and LV mass index below the normal reference range.^20^ Overall, RV mass index, RV volumes at ED (RV EDVi) and ES (RV ESVi), and RV mass/volume ratio were significantly higher in patients with ToF compared with healthy controls (Table 2). LV EF, LV mass index, LV mass/volume ratio, and LV/RV mass ratio were decreased in patients with ToF, and LV ESVi was higher than in healthy controls. LV EDVi did not differ between the ToF group and the control group. LV EDVi correlated with RV EDVi (*r*=0.36, *P*<0.001). Patients with ToF and controls had similar heart rates (*P*=0.115; Table 1). In patients with ToF and healthy controls, LV volumes and mass correlated strongly with age, and the correlations remained moderate to strong if these LV parameters were indexed by BSA. For each LV parameter, Table 3 lists the proportion of patients with ToF with values outside the corresponding *age‐adjusted* 95% confidence interval for healthy controls. For example, despite the fact that mean LV EDVi was not significantly different between patients with ToF and controls, 34% of patients with ToF had an LV EDVi above the age‐adjusted 95th percentile value.

**Table 2:** Comparison of CMR Measurements in Patients With ToF and Controls

Variable	Patients With ToF (n=103)	Controls (n=63)	*P* Value
LV EF, %	52.2±8.0	59.0±5.4	<0.01
LV SVi, mL/m^2^	41.2±11.4	45.2±7.5	<0.01
LV EDVi, mL/m^2^	80.5±20.6	77.2±11.5	0.6
LV ESVi, mL/m^2^	39.2±13.5	32.8±8.6	<0.01
LV mass index, g/m^2^	45.5±12.5	49.6±9.23	<0.01
LV mass/volume ratio	0.58±0.13	0.65±0.13	<0.01
LV cardiac output index, L/min per m^2^	3.0±0.1	3.3±0.1	0.05
LV/RV mass ratio	1.47±0.54	2.54±0.49	<0.01
LV ECV, ×10	3.2±0.05 (n=57)	2.63±0.01	<0.01
RV EF, %	44.0±9.94	46.6±12.7	0.36
RV EDVi, mL/m^2^	120.0±34.4	82.7±15.4	<0.01
RV ESVi, mL/m^2^	69.0±24.9	43.8±13.5	<0.01
RV mass index, g/m^2^	34.1±12.6	20.2±5.6	<0.01
RV mass/volume ratio	0.29±0.11	0.25±0.05	0.03
P‐RGF, %	28.3±17.1	…	…
LA_max_ index, mL/m^2^	32.4±12.6 (n=86)	41.7±10.2	<0.01
LA_min_ index, mL/m^2^	16.5±7.9 (n=86)	18.3±5.4	0.013
LA contractile volume index, mL/m^2^	5.97±3.55 (n=86)	6.35±2.7	0.25
LA emptying passive volume index, mL/m^2^	9.97±4.56 (n=86)	17.0±5.0	<0.01
LA PEF, %	31.7±11.0	40.7±7.4	<0.01
LA CEF, %	26.8±9.99	25.6±8.6	0.50
LA EF, %	50.2±9.4	56.0±6.96	<0.01

Data are presented as mean±SD. *P* values are from the Wilcoxon or Fisher exact test. CEF indicates contractile emptying fraction; CMR, cardiac magnetic resonance; ECV, extracellular volume; EDVi, end‐diastolic volume index; EF, ejection fraction; ESVi, end‐systolic volume index; LA, left atrial; LA_max_ index, maximum LA volume indexed by body surface area; LA_min_ index, minimum left atrial volume indexed by body surface area; LV, left ventricular; PEF, passive emptying fraction; P‐RGF, pulmonary regurgitation fraction; RV, right ventricular; SVi, stroke volume index; ToF, tetralogy of Fallot.

**Table 3:** Proportions of ToF Outside the Normal Range for LV and LA Parameters

Parameter	Proportion for <5th or >95th Percentiles	5%–95% CI
LV EF <5th percentile	0.49	0.39–0.60
LV EDVi >95th percentile	0.34	0.25–0.44
LV ESVi >95th percentile	0.23	0.15–0.32
LV mass index <5th percentile	0.28	0.20–0.38
LV mass/volume ratio <5th percentile	0.28	0.17–0.35
LA EF passive <5th percentile	0.38	0.28–0.49
LV ECV >95th percentile	0.66	0.52–0.78

The 5% to 95% CI in the right‐hand column refers to the CI for the proportion (not the actual number of the measurement). ECV indicates extracellular volume; EDVi, end‐diastolic volume index; EF, ejection fraction; ESVi, end‐systolic volume index; LA, left atrial; LV, left ventricular; ToF, tetralogy of Fallot.

**Figure 2:**
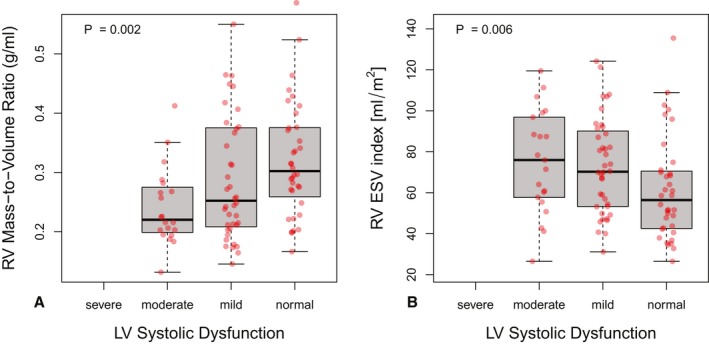
Left ventricular (LV) systolic dysfunction in patients with tetralogy of Fallot ToF. **A**, The right ventricular (RV) mass/volume ratio was significantly lower in patients with ToF and LV systolic dysfunction. LV dysfunction was defined, following the categorization of Broberg et al,^21^ as severe (LV ejection fraction [EF] <35%), moderate (35% <LV EF <45%), or mild (45% <LV EF <55%). **B**, The RV end‐systolic volume (ESV) index increased from normal to moderate LV systolic dysfunction. The trends across the categories of systolic dysfunction in **A** and **B** were examined with the nonparametric Jonckheere test.

In ToF, LV EF correlated with RV EF (ρ=0.41; *P*<0.001; 95% CI, 0.26–0.54) and RV mass/volume ratio (ρ=0.33; *P*<0.001). Using the categories of LV systolic dysfunction used in the study of patients with ToF by Broberg et al,^21^ we observed no cases of severe dysfunction (LV EF <35%), 23% of cases showed moderate dysfunction (35% <LV EF <45%), 41% had mild dysfunction (45% <LV EF <55%), and 36% had normal LV EF. Figure 2A shows how the degree of LV systolic dysfunction was associated with remodeling of the RV, quantified by the RV mass/volume ratio (*P*=0.002 for Jonckheere nonparametric test for increasing RV mass/volume ratio across ordered categories of LV systolic dysfunction). The association between LV systolic dysfunction and RV mass/volume ratio remained significant in a multivariate model for RV mass/volume ratio, with LV EF and age (*P*=0.33 for age effect) as simultaneous independent predictors. Figure 2B shows how LV systolic dysfunction is associated with RV ESVi, a marker of systolic function that is potentially less prone to confounding by RV volume overloading than RV EF. Nevertheless, RV EF (not shown in Figure 2) also decreased between normal through mild to moderate LV dysfunction (*P*=0.002 for Jonckheere nonparametric test). The probability of less than mild LV dysfunction decreased by 0.38:1 for a 0.1 increase of the RV mass/volume ratio (*P*=0.009).

**Figure 3:**
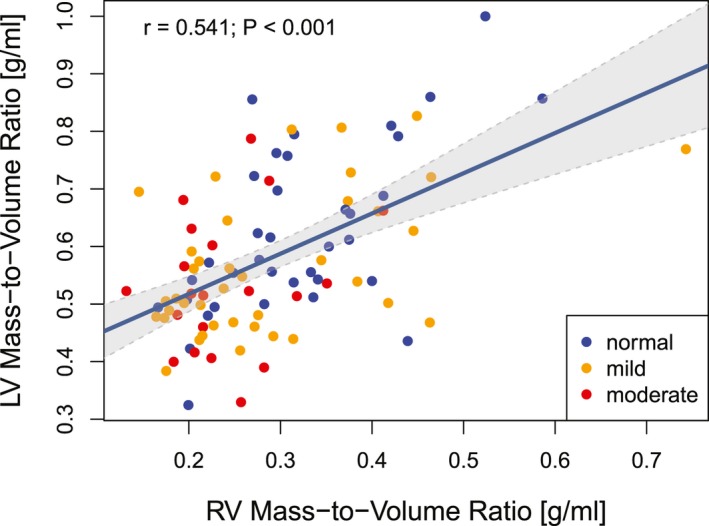
Remodeling of the left ventricle (LV), quantified by the LV mass/volume ratio, correlated with the right ventricular (RV) mass/volume ratio (*r*=0.054; *P*<0.001) in patients with tetralogy of Fallot (ToF). Moderate LV systolic dysfunction, identified by red data points, was associated with a lower RV mass/volume ratio, whereas normal LV systolic function (blue circles) predominated at higher levels of RV mass/volume ratio and LV mass/volume ratio. The continuous line represents the least squares regression line, with gray‐shaded area showing the 95th percentile prediction interval.

**Figure 4:**
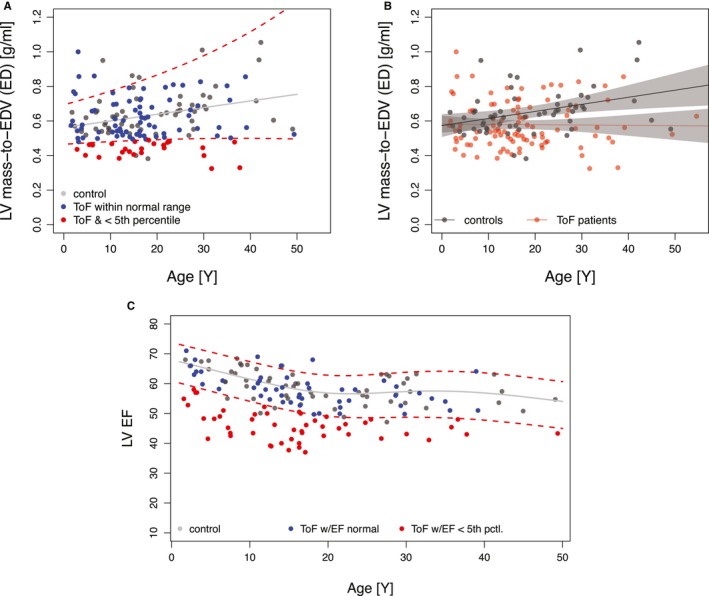
**A**, The median and 5^th^ and 95th percentile levels for left ventricular (LV) mass/volume ratio, a marker of LV global remodeling, are represented by gray and dashed red lines, respectively, for healthy controls, and were determined with a generalized additive model for location, shape, and scale. With age‐specific percentile levels, 28% of patients with tetralogy of Fallot (ToF) had an LV mass/volume ratio <5th percentile in healthy controls (red filled circles), and define a subgroup with eccentric remodeling. Patients with ToF with LV mass/volume ratio >5th percentile level in controls are shown as blue filled circles. A similar analysis was performed for other cardiac magnetic resonance–derived LV parameters to determine the proportions of patients with ToF with values outside the 90th percentile range, summarized in Table 3. One data point for a patient aged 55 years is not shown in **A** because the data for normal volunteers lacked sufficient density above ≈50 years of age to determine a 95^th^ percentile range. **B**, Linear regression analysis showed that, in healthy controls, LV mass/volume ratio increased significantly with age (*P*=0.007), whereas in patients with ToF, there was no significant association with age. The lines represent the predicted mean from the linear regression model, and the gray bands represent their 95% CIs. **C**, The median and 5th and 95th percentile levels for LV ejection fraction (EF) in healthy controls, shown as gray and dashed red lines, respectively, changed significantly with age. With age‐specific percentile levels, 49% of patients with ToF had an LV mass/volume ratio <5th percentile in healthy controls (red circles). Patients with ToF and LV EF >5th percentile level in controls are shown as blue filled circles. ED indicates end diastole; EDV, ED volume.

LV mass/volume ratio, a parameter characterizing LV remodeling, was significantly lower in patients with ToF than in healthy controls (0.58±0.13 versus 0.65±0.13 g/mL; *P*=0.001). LV mass/volume ratio correlated with RV mass/volume ratio (*r*=0.54; *P*<0.001), as shown in Figure 3, and RV mass index (*r*=0.42; *P*<0.001). Approximately 28% of all patients with ToF had a LV mass/volume ratio below the age‐specific fifth percentile level in healthy controls, as illustrated in Figure 4.

**Figure 5:**
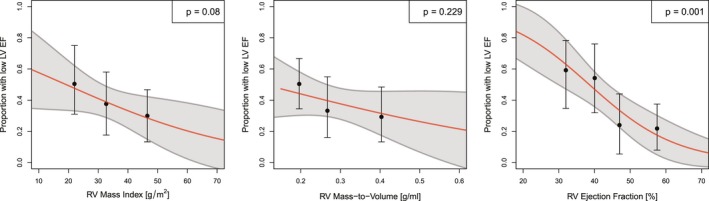
The proportion of patients with tetralogy of Fallot (ToF) with a left ventricular (LV) mass/volume ratio below the age‐adjusted 5th percentile in healthy controls is shown for each quartile (solid circles) of right ventricular (RV) mass index (**A**), RV mass/volume ratio (**B**), and RV ejection fraction (EF) (**C**), with error bars denoting the 95th percentile ranges obtained by the bootstrap method. The solid red lines (with gray bands representing the 5th to 95th percentile prediction limits) show the best fit for logistic regression models for low LV mass/volume ratio (<5th percentile in controls) with RV mass index (**A**), RV mass/volume ratio (**B**), and RV EF (**C**) as predictors. In **A**, the odds of an abnormally low LV mass/volume ratio were predicted to decrease by 0.53:1 for a 10‐g/m² increase of RV mass index (*P*=0.013); and in **B**, the odds decreased by 0.39:1 for each 0.1‐g/mL increase in RV mass/volume ratio (*P*=0.007). **C**, In contrast, RV EF was not a significant predictor.

The probability of abnormally low LV mass/volume ratio decreased by 0.53:1 for a 10‐g/m² increase of RV mass index (*P*=0.013; Figure 5A) and also by 0.39:1 for each 0.1‐g/mL increase in RV mass/volume ratio (*P*=0.007; Figure 5B). There was no significant association between abnormal LV mass/volume ratio and RV EF (*P*=0.225; Figure 5C).

### Atrial Volumes

LA volumes and function were decreased compared with controls (Table 2). LA_max_, a reflection of LV filling pressures, correlated with age, age at surgical repair, and LV volume indexes (*P*<0.01). Total and passive LA EFs were negatively associated with age, unlike in healthy controls, in whom they remained nearly constant.

### Focal Fibrosis and ECV Fraction

LGE imaging was performed in 65 patients: 62 (95%) of those had LGE at the site of ventricular septal defect patching, 30 (46.1%) showed LGE in the RV outflow tract, and 9 (13.8%) had small spots of LGE that were detected in other areas of the heart but not the LV free wall.

Myocardial ECV was obtained in 57 (55%) of 103 patients with ToF, from the time when the T1 sequence was available on the scanner. It was significantly elevated compared with controls (0.32±0.05 versus 0.26±0.01; *P*<0.01). Female patients with ToF showed a significantly higher ECV than male patients (*P*=0.033). Even when the patients with ToF and LGE in the LV were excluded, ECV was still significantly higher compared with controls (0.32±0.06 in patients with ToF versus 0.26±0.01 in controls; *P*<0.01). For patients with ToF and abnormally low LV EF, ECV was significantly higher in the septum, compared with patients with ToF and LV EF within the normal range (*P*=0.02). The LV mass index correlated negatively with ECV (*r*=−0.32; *P*=0.016).

### Cardiopulmonary Exercise Testing

CPET results were available for 70 patients with ToF (68%) who reached the anaerobic threshold. VO_2_ max was subnormal (lower than the third percentile for reference values in normal population) in 20 patients (29%), and there was no significant difference in the proportion of abnormal VO_2_ max between male and female patients with ToF (21% for male patients and 39% for female patients; *P*=0.16).

**Figure 6:**
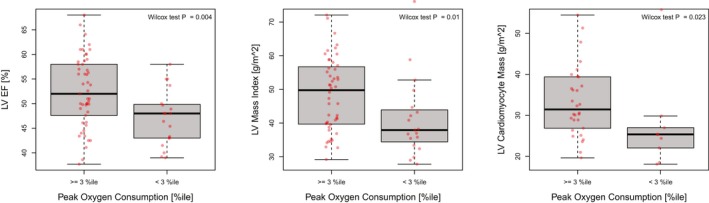
A subnormal peak oxygen consumption (VO_2_ max) in patients with tetralogy of Fallot (ToF) (n=70; 68%), defined as VO_2_ max below the third percentile level of age‐matched healthy people, was associated with lower resting left ventricular (LV) systolic dysfunction (**A**), lower LV mass index (**B**), and a lower LV cardiomyocyte mass index (LV mass index · [1‐extracellular volume (ECV)]) (**C**), suggesting atrophy. ECV was available in a subset of 57 patients with ToF (55%). EF indicates ejection fraction.

In patients with ToF and abnormally low VO_2_ max, impaired LV resting systolic function and adverse LV remodeling were more frequent than in patients with VO_2_ max above the third percentile level for controls. In univariate logistic regression models, the likelihood of an abnormally low VO_2_ max increased with lower LV EF (odds ratio of 1.13 per 1% decrease of LV EF; *P*=0.008) and a lower LV mass index (odds ratio of 1.9:1 for each 10‐g/m² decrease of LV mass index; *P*=0.024). LV EF and LV mass index remained significant predictors of an abnormally low VO_2_ max, when RV mass/volume ratio, RV ESV index, and RV EF were each added as additional predictor in these 2 models, suggesting that LV dysfunction increases the likelihood of impaired exercise capacity, *independent* of the degree of RV systolic dysfunction and remodeling. A comparison of the patients with ToF and abnormally low VO_2_ max and normal VO_2_ max showed that they had a lower LV EF (Figure 6A), a lower LV mass index (Figure 6B), and a lower cardiomyocyte mass index, resulting from a higher ECV fraction (Figure 6C). To further corroborate our results, we calculated 2 ECV averages for each patient: one for the intraventricular septum and one for the LV free wall (the latter is definitely representative of ECV in segments without any nearby LGE). Herein, we see also a significant (*P*=0.02) difference for the free wall ECV average between patients with ToF with normal and abnormal VO_2_ max.

**Figure 7:**
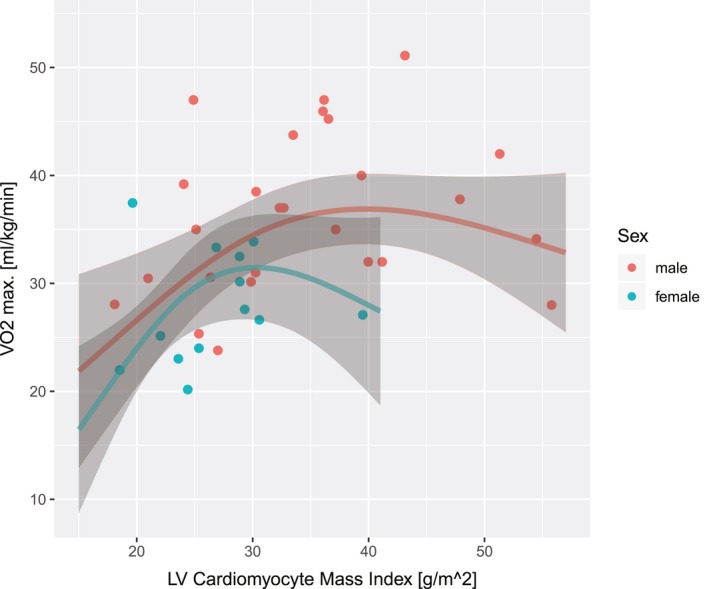
Peak oxygen consumption (VO_2_ max), measured in patients with tetralogy of Fallot (ToF) during cardiopulmonary exercise testing (in mL/kg per minute), was modeled with a generalized additive model (GAM) as a function of age, sex, left ventricular (LV) ejection fraction, and LV cardiomyocyte mass index (CMi) (ie, the univariate predictors, other than age and sex, that had a significant effect on abnormal exercise capacity) (see Figure 6). The dependence of VO_2_ max on CMi (*P*=0.015) predicted by this GAM is shown for male and female sex by the continuous lines for male and female patients with ToF, respectively. VO_2_ max declined for CMi <≈39 g/m^2^ in male patients and CMi <≈30 g/m^2^ in female patients, demonstrating the effect of LV atrophy in ToF on exercise capacity, independent of age, sex, and systolic LV function. Age, LV ejection fraction, and cardiomyocyte mass were represented as cubic regression splines. The GAM model for VO_2_ max explained 51% of the observed deviance. The gray bands show the 95% CIs.

In a multivariate generalized additive model for VO_2_ max (in mL/kg per minute), the cardiomyocyte mass had a significant association with VO_2_ max (*P*=0.014), with simultaneous adjustment by sex (*P*=0.06), age (*P*=0.25), and LV EF (*P*=0.057). The predicted dependence of VO_2_ max on cardiomyocyte mass index is shown in Figure 7 as continuous lines for male and female sex, with gray‐shaded 95% confidence bands around each line.

## Discussion

In this study, clinically asymptomatic patients with repaired ToF frequently show left‐sided diastolic and systolic dysfunction and a lower than normal LV mass/volume ratio that is consistent with a previously described pattern of eccentric LV remodeling.^9^, ^10^, ^22^ Adverse changes in the RV, such as a *lower*‐than‐average RV mass, RV dilation (ie, higher RV EDVi), and a *lower* RV mass/volume ratio, are associated with a lower LV mass/volume ratio. The diminished LV mass observed in ToF, in combination with the expansion of the ECV in the LV, implies a loss of total cardiomyocyte mass indicative of LV atrophy. LV systolic dysfunction and the loss of LV mass correlated with impaired exercise tolerance, independently of the RV parameters that predict LV remodeling (RV mass/volume ratio) and LV dysfunction (RV ESVi and RV EF). In a multivariate analysis, LV atrophy had a stronger effect on VO_2_ max than LV EF, suggesting that LV cardiomyocyte mass may be an important parameter for monitoring LV remodeling in patients with ToF, with the advantage, compared with LV EF, that it is not dependent on hemodynamic loading conditions. In light of previous studies,^4^, ^23^, ^24^ showing that adverse changes in the LV may contribute to the overall risk of adverse clinical outcomes in ToF, our results suggest that surveillance of LV remodeling in ToF, starting in clinically asymptomatic patients, may provide an early therapeutic window to prevent progression to clinically overt LV dysfunction and failure.^24^

### ECV and Loss of Cardiomyocyte Mass

Histological studies have shown increased interstitial collagen content in both the RV and LV walls of patients with ToF.^25^, ^26^ ECV is a noninvasive surrogate marker of diffuse interstitial fibrosis, which, similarly to the histological studies, indicates that diffuse interstitial fibrosis is an important pathophysiological feature of myocardial tissue remodeling in ToF.^12^, ^27^ In our study, female sex was an independent predictor of elevated ECV in ToF, consistent with the previous finding^28^ that female patients with ToF are more likely than male patients to show a cardiomyopathic phenotype with LV dilatation.

ECV was negatively associated with the LV mass index, suggesting that interstitial fibrosis is linked with LV atrophy in ToF. The negative correlation in ToF of ECV with the LV mass index is opposite to a positive correlation observed in pathological hypertrophy resulting from hypertensive heart disease^29^ or aortic stenosis,^13^ despite the fact that in both cases ECV is similarly increased. The key differentiator between these 2 forms of extracellular remodeling is the loss of LV cardiomyocyte mass, which can be estimated from the product of (1‐ECV), the cardiomyocyte fraction of the tissue, and LV mass. An increase of the ECV fraction, or analogously a decrease of (1‐ECV), if not compensated by an increase of LV mass, results in a net decrease of the LV cardiomyocyte mass. In patients with ToF, a below normal LV mass and an ECV expansion both contribute to a net loss of cardiomyocyte mass. The functional and potential clinical significance of these findings is highlighted by the fact that ECV was higher in the patients with ToF with less exercise tolerance (Figure 6A through 6C). The underlying mechanisms for a loss of total cardiomyocyte mass or LV atrophy in ToF are not well understood but may be related to reduced LV diastolic filling rates observed in patients with ToF.^30^, ^31^, ^32^, ^33^ Analogously, LV atrophy was observed as a correlate of impaired diastolic filling in an animal model of RV failure^34^ and in patients with RV failure, secondary to chronic thromboembolic pulmonary hypertension.^35^

### Systolic and Diastolic Dysfunction

Approximately two thirds of patients with ToF had mild‐to‐moderate LV systolic dysfunction, and in nearly half of them the LV EF was below the age‐adjusted fifth percentile for normal controls (Table 3). This is a significantly higher proportion than the 21% prevalence reported in a previous echocardiographic study^21^ that did not use age‐adjusted percentile curves (this may result in an underestimation of the prevalence of impaired LV function in younger patients with ToF, in whom the fifth percentile level for LV EF in healthy controls reaches 55%, rather than the customary adult cutoff of 50% or 45%).^36^, ^37^

The subclinical LV abnormalities reported in our study (eg, increase of ECV and LV ESVi and eccentric remodeling) may contribute to the reported risk of later clinical left‐sided heart failure and cardiac death in ToF.^4^, ^21^, ^23^, ^38^ LV systolic dysfunction was associated in our patients with ToF with impaired exercise capacity (Figure 6), independent of markers of RV systolic dysfunction (RV ES volume and RV mass/volume ratio). Specifically, with the statistical model that predicts an abnormal exercise capacity from LV EF, any RV parameter added to the model did not have a significant effect and, furthermore, did not change significantly the effect LV EF. This suggests that LV dysfunction may not be simply a mechanical corollary of RV dysfunction, like a correlation between RV EF and LV EF, but is compounded by cardiomyopathic processes in the LV, characterized by atrophy and an increase of ECV. Figure 7 illustrates that the decline of VO_2_ max sets in for LV cardiomyocyte mass indexes <≈39 g/m^2^ in male patients and <≈30 g/m^2^ in female patients, indicative for LV atrophy. For comparison, assume a healthy 20‐year‐old man with a myocardial ECV fraction of 0.25 (eg, the study by Broberg et al,^38^ with an ECV of 24.8±2.0% for healthy controls) and a 95% CI for LV mass index from 60 to 93 g/m^2^: the resulting 95% confidence range for LV cardiomyocyte mass index, calculated as (1−ECV)×LV mass index, would range from ≈45 to 70 g/m^2^. The observed decline of VO_2_ max around a level of 39 g/m^2^ is, therefore, consistent with the expected threshold for LV atrophy on the basis of published reference values for LV mass index. A higher ECV, indicative of diffuse fibrosis, would lower this threshold further. The investigated parameters of LV dysfunction (eg, LV ESVi and LV EF) may, therefore, have a functional significance for predicting impaired exercise capacity that goes beyond the changes in RV volumes and function. In the statistical analysis, the significance of LV EF for prediction of impaired exercise capacity remained with adjustment by RV parameters, equally suggesting that LV EF in this context should not be interpreted as surrogate for RV markers of dysfunction. In contrast to previous studies,^3^, ^38^, ^39^ only a moderate but highly significant (*P*<0.001) correlation between RV EF and LV EF was observed in our study (ρ=0.41 versus ρ=0.58 in Geva et al^38^). The stronger correlation between RV and LV EFs in the study by Geva et al^38^ may be caused by the relatively high proportion of patients with ToF in functional class of New York Heart Association >1 (52%), whereas our cohort only included asymptomatic patients with ToF. In adults with acquired heart disease and compromised RV EF,^40^ the correlation between RV and LV EF is noticeably stronger, compared with patients with normal biventricular function. Similarly, Broberg et al showed that LV systolic dysfunction is more prevalent in patients with ToF and moderate to severe RV dysfunction, compared with those with mild RV dysfunction.^21^ As RV EF is prone to confounding by pulmonary insufficiency and volume overloading in ToF,^28^, ^41^ we determined which other RV parameters correlated with left‐sided heart remodeling and dysfunction: both RV mass/volume ratio and RV ESVi, a marker of RV systolic function, were predictors of LV remodeling (LV mass/volume ratio) and systolic dysfunction (LV EF), independent of sex and age.

Valente et al previously investigated in patients with ToF the association between markers of RV and LV remodeling/dysfunction and outcome (death and ventricular tachycardia).^4^ Their best multivariate predictive model for death and ventricular tachycardia within a median follow‐up time after magnetic resonance imaging of 1.9 years included as predictors poor LV systolic function (LV EF *z* score <−2.0), an RV mass/volume ratio ≥0.3 g/mL, and history of atrial tachyarrhythmias; however, more important, RV EF was not a predictor. These findings in an older population (median age, 24 versus 16 years) with signs of overt heart failure underscore the potential importance of LV systolic dysfunction for adverse outcomes. Bokma et al^42^ more recently presented a prediction model from a retrospective multicenter study of 575 patients with ToF (mean age, 31 years) from which LV EF and RV EF emerged as the 2 strongest outcome predictors. The results suggest that the at‐risk LV in ToF should be monitored for subclinical signs of adverse remodeling and dysfunction.

Diastolic dysfunction may be one of the earliest manifestations of adverse LV remodeling in ToF. We examined LA function by CMR cine volumetry,^11^ as a surrogate marker for LV diastolic function. Passive and total LA EFs, as well as the LA volumes, were decreased in our ToF cohort (Table 2), which is in line with the echocardiographic findings of Koenigstein et al^31^ in a cohort of 25 asymptomatic patients with ToF. In another CMR study of 20 patients with ToF, Riesenkampff et al^33^ also found diminished LA volumes as a sign of pulmonary underfilling and lower available preload.

### Limitations

The results of this study should be viewed in the context of its design. Given that this is a cross‐sectional study, we cannot relate causes and effects in the interplay of LV function and structure, intrinsic tissue‐level alterations (by ECV), RV‐LV interdependence, diastolic properties (as reflected by LA function), or vascular stiffness. Patients aged <7 years (15% of ToF cohort) were sedated during the CMR studies, which may have depressed cardiac function. The statistical significance of the main findings (eg, Figures 2, 3, 4, 5, 6 through 7) did not materially change if the analysis was repeated after exclusion of CMR studies with sedation. The potential implications of our study in terms of optimal timing for surgical intervention or medical therapy for prevention of heart failure, arrhythmias, and sudden cardiac death need to be investigated in larger, longitudinal studies. Further limitations are missing data for LGE (40%) and ECV (45% of patients with ToF), as patients or their guardians declined contrast, and T1 mapping became available on the scanner only during the course of the study. Cardiopulmonary exercise testing results were not available for 33 (32%) of patients, mostly because of young age, refusal of the test, or not reaching the anaerobic threshold.

The availability of CPET for only a subset of 70 (68%) of patients with ToF may have introduced a selection bias in the analysis of the associations between abnormally low VO_2_ max and LV parameters. Patients with ToF with CPET were older (19.8±10.4 versus 11.4±8.85 years for patients with ToF without CPET) and had a lower body mass index (18.4±4.82 versus 21.9±4.7 kg/m^2^), but showed no difference in sex distribution.

### Conclusions

This multiparametric CMR study on LV remodeling in asymptomatic patients with ToF at a median of 14 years after surgical repair revealed the following: (1) frequent (49%) impaired systolic function over the full age range, abnormal diastolic relaxation, a decreased LV mass, and interstitial matrix expansion (as reflected by ECV), which are all evidence of adverse LV remodeling and a cardiomyopathic phenotype (the best predictor for LV systolic dysfunction was the RV mass/volume ratio); and (2) CMR markers of LV systolic dysfunction and adverse tissue remodeling (reduced cardiomyocyte mass) correlated with subnormal VO_2_ max, an important risk predictor in ToF.

Therefore, early surgical reintervention for pulmonary insufficiency if present, or medical heart failure therapy, both guided by CMR‐guided surveillance of the LV, may delay progression of adverse remodeling and improve the long‐term prognosis.

## Sources of Funding

This work was supported by a postdoctoral fellowship grant (6619‐10‐0) for Dr Andrade from the Coordination of Improvement of Higher Education Personnel of Brazil and by a research grant from the foundation “Kinderherzen–wollen‐leben e.V.” for Dr Pham (http://www.kinderherzen-wollen-leben.de/).

## Disclosures

None.

## References

[1] Geva T . Repaired tetralogy of Fallot: the roles of cardiovascular magnetic resonance in evaluating pathophysiology and for pulmonary valve replacement decision support. J Cardiovasc Magn Reson. 2011;13:9. 21251297 10.1186/1532-429X-13-9 PMC3036629

[2] Cuypers JA , Menting ME , Konings EE , Opic P , Utens EM , Helbing WA , Witsenburg M , van den Bosch AE , Ouhlous M , van Domburg RT , Rizopoulos D , Meijboom FJ , Boersma E , Bogers AJ , Roos‐Hesselink JW . Unnatural history of tetralogy of Fallot: prospective follow‐up of 40 years after surgical correction. Circulation. 2014;130:1944–1953. 25341442 10.1161/CIRCULATIONAHA.114.009454

[3] Davlouros PA , Kilner PJ , Hornung TS , Li W , Francis JM , Moon JC , Smith GC , Tat T , Pennell DJ , Gatzoulis MA . Right ventricular function in adults with repaired tetralogy of Fallot assessed with cardiovascular magnetic resonance imaging: detrimental role of right ventricular outflow aneurysms or akinesia and adverse right‐to‐left ventricular interaction. J Am Coll Cardiol. 2002;40:2044–2052. 12475468 10.1016/s0735-1097(02)02566-4

[4] Valente AM , Gauvreau K , Assenza GE , Babu‐Narayan SV , Schreier J , Gatzoulis MA , Groenink M , Inuzuka R , Kilner PJ , Koyak Z , Landzberg MJ , Mulder B , Powell AJ , Wald R , Geva T . Contemporary predictors of death and sustained ventricular tachycardia in patients with repaired tetralogy of Fallot enrolled in the indicator cohort. Heart. 2014;100:247–253. 24179163 10.1136/heartjnl-2013-304958 PMC3913216

[5] Kawel‐Boehm N , Maceira A , Valsangiacomo‐Buechel ER , Vogel‐Claussen J , Turkbey EB , Williams R , Plein S , Tee M , Eng J , Bluemke DA . Normal values for cardiovascular magnetic resonance in adults and children. J Cardiovasc Magn Reson. 2015;17:29. 25928314 10.1186/s12968-015-0111-7 PMC4403942

[6] Broberg CS , Chugh SS , Conklin C , Sahn DJ , Jerosch‐Herold M . Quantification of diffuse myocardial fibrosis and its association with myocardial dysfunction in congenital heart disease. Circ Cardiovasc Imaging. 2010;3:727–734. 20855860 10.1161/CIRCIMAGING.108.842096 PMC3048790

[7] Muller J , Hager A , Diller GP , Derrick G , Buys R , Dubowy KO , Takken T , Orwat S , Inuzuka R , Vanhees L , Gatzoulis M , Giardini A . Peak oxygen uptake, ventilatory efficiency and QRS‐duration predict event free survival in patients late after surgical repair of tetralogy of Fallot. Int J Cardiol. 2015;196:158–164. 26114442 10.1016/j.ijcard.2015.05.174

[8] Kramer CM , Barkhausen J , Flamm SD , Kim RJ , Nagel E ; Society for Cardiovascular Magnetic Resonance Board of Trustees Task Force on Standardized Protocols . Standardized cardiovascular magnetic resonance imaging (CMR) protocols, society for cardiovascular magnetic resonance: board of trustees task force on standardized protocols. J Cardiovasc Magn Reson. 2008;10:35. 18605997 10.1186/1532-429X-10-35 PMC2467420

[9] Gaasch WH , Zile MR . Left ventricular structural remodeling in health and disease: with special emphasis on volume, mass, and geometry. J Am Coll Cardiol. 2011;58:1733–1740. 21996383 10.1016/j.jacc.2011.07.022

[10] Garg S , de Lemos JA , Ayers C , Khouri MG , Pandey A , Berry JD , Peshock RM , Drazner MH . Association of a 4‐tiered classification of LV hypertrophy with adverse CV outcomes in the general population. JACC Cardiovasc Imaging. 2015;8:1034–1041. 26298074 10.1016/j.jcmg.2015.06.007 PMC4575632

[11] Kaminski M , Steel K , Jerosch‐Herold M , Khin M , Tsang S , Hauser T , Kwong RY . Strong cardiovascular prognostic implication of quantitative left atrial contractile function assessed by cardiac magnetic resonance imaging in patients with chronic hypertension. J Cardiovasc Magn Reson. 2011;13:42. 21843343 10.1186/1532-429X-13-42 PMC3195715

[12] Chen CA , Dusenbery SM , Valente AM , Powell AJ , Geva T . Myocardial ECV fraction assessed by CMR is associated with type of hemodynamic load and arrhythmia in repaired tetralogy of Fallot. JACC Cardiovasc Imaging. 2016;9:1–10. 26684969 10.1016/j.jcmg.2015.09.011

[13] Dusenbery SM , Jerosch‐Herold M , Rickers C , Colan SD , Geva T , Newburger JW , Powell AJ . Myocardial extracellular remodeling is associated with ventricular diastolic dysfunction in children and young adults with congenital aortic stenosis. J Am Coll Cardiol. 2014;63:1778–1785. 24632273 10.1016/j.jacc.2013.11.066

[14] Jerosch‐Herold M , Sheridan DC , Kushner JD , Nauman D , Burgess D , Dutton D , Alharethi R , Li D , Hershberger RE . Cardiac magnetic resonance imaging of myocardial contrast uptake and blood flow in patients affected with idiopathic or familial dilated cardiomyopathy. Am J Physiol Heart Circ Physiol. 2008;295:H1234–H1242. 18660445 10.1152/ajpheart.00429.2008 PMC2544489

[15] Dubowy KO , Baden W , Bernitzki S , Peters B . A practical and transferable new protocol for treadmill testing of children and adults. Cardiol Young. 2008;18:615–623. 18838025 10.1017/S1047951108003181

[16] Möller P , Weitz M , Jensen KO , Dubowy KO , Furck AK , Scheewe J , Kramer HH , Uebing A . Exercise capacity of a contemporary cohort of children with hypoplastic left heart syndrome after staged palliation. Eur J Cardiothorac Surg. 2009;36:980–985. 19643621 10.1016/j.ejcts.2009.06.029

[17] Kempny A , Dimopoulos K , Uebing A , Moceri P , Swan L , Gatzoulis MA , Diller GP . Reference values for exercise limitations among adults with congenital heart disease relation to activities of daily life: single centre experience and review of published data. Eur Heart J. 2012;33:1386–1396. 22199119 10.1093/eurheartj/ehr461

[18] R Core Team . R: A Language and Environment for Statistical Computing. Vienna, Austria: R Foundation for Statistical Computing; 2015 https://www.R-project.org. Accessed November 11, 2015.

[19] Dabir D , Child N , Kalra A , Rogers T , Gebker R , Jabbour A , Plein S , Yu CY , Otton J , Kidambi A , McDiarmid A , Broadbent D , Higgins DM , Schnackenburg B , Foote L , Cummins C , Nagel E , Puntmann VO . Reference values for healthy human myocardium using a T1 mapping methodology: results from the international T1 multicenter cardiovascular magnetic resonance study. J Cardiovasc Magn Reson. 2014;16:69. 25384607 10.1186/s12968-014-0069-x PMC4203908

[20] Maceira AM , Prasad SK , Khan M , Pennell DJ . Normalized left ventricular systolic and diastolic function by steady state free precession cardiovascular magnetic resonance. J Cardiovasc Magn Reson. 2006;8:417–426. 16755827 10.1080/10976640600572889

[21] Broberg CS , Aboulhosn J , Mongeon FP , Kay J , Valente AM , Khairy P , Earing MG , Opotowsky AR , Lui G , Gersony DR , Cook S , Ting JG , Webb G , Gurvitz MZ ; Alliance for Adult Research in Congenital Cardiology (AARCC) . Prevalence of left ventricular systolic dysfunction in adults with repaired tetralogy of Fallot. Am J Cardiol. 2011;107:1215–1220. 21349477 10.1016/j.amjcard.2010.12.026

[22] Li SN , Yu W , Lai CT , Wong SJ , Cheung YF . Left ventricular mechanics in repaired tetralogy of Fallot with and without pulmonary valve replacement: analysis by three‐dimensional speckle tracking echocardiography. PLoS One. 2013;8:e78826. 24223166 10.1371/journal.pone.0078826 PMC3819374

[23] Diller GP , Kempny A , Liodakis E , Alonso‐Gonzalez R , Inuzuka R , Uebing A , Orwat S , Dimopoulos K , Swan L , Li W , Gatzoulis MA , Baumgartner H . Left ventricular longitudinal function predicts life‐threatening ventricular arrhythmia and death in adults with repaired tetralogy of Fallot. Circulation. 2012;125:2440–2446. 22496160 10.1161/CIRCULATIONAHA.111.086983

[24] Knauth AL , Gauvreau K , Powell AJ , Landzberg MJ , Walsh EP , Lock JE , del Nido PJ , Geva T . Ventricular size and function assessed by cardiac MRI predict major adverse clinical outcomes late after tetralogy of Fallot repair. Heart. 2008;94:211–216. 17135219 10.1136/hrt.2006.104745

[25] Farah MC , Castro CR , Moreira VM , Riso Ade A , Lopes AA , Aiello VD . The myocardium in tetralogy of Fallot: a histological and morphometric study. Arq Bras Cardiol. 2009;92:160‐7–163‐71. 19390702 10.1590/s0066-782x2009000300002

[26] Jeewa A , Manickaraj AK , Mertens L , Manlhiot C , Kinnear C , Mondal T , Smythe J , Rosenberg H , Lougheed J , McCrindle BW , van Arsdell G , Redington AN , Mital S . Genetic determinants of right‐ventricular remodeling after tetralogy of Fallot repair. Pediatr Res. 2012;72:407–413. 22797143 10.1038/pr.2012.95

[27] Riesenkampff E , Luining W , Seed M , Chungsomprasong P , Manlhiot C , Elders B , McCrindle BW , Yoo SJ , Grosse‐Wortmann L . Increased left ventricular myocardial extracellular volume is associated with longer cardiopulmonary bypass times, biventricular enlargement and reduced exercise tolerance in children after repair of tetralogy of Fallot. J Cardiovasc Magn Reson. 2016;18:75. 27782857 10.1186/s12968-016-0290-x PMC5080785

[28] Sarikouch S , Koerperich H , Dubowy KO , Boethig D , Boettler P , Mir TS , Peters B , Kuehne T , Beerbaum P ; German Competence Network for Congenital Heart Defects Investigators . Impact of gender and age on cardiovascular function late after repair of tetralogy of Fallot: percentiles based on cardiac magnetic resonance. Circ Cardiovasc Imaging. 2011;4:703–711. 21908707 10.1161/CIRCIMAGING.111.963637

[29] Kuruvilla S , Janardhanan R , Antkowiak P , Keeley EC , Adenaw N , Brooks J , Epstein FH , Kramer CM , Salerno M . Increased extracellular volume and altered mechanics are associated with LVH in hypertensive heart disease, not hypertension alone. JACC Cardiovasc Imaging. 2015;8:172–180. 25577446 10.1016/j.jcmg.2014.09.020 PMC4418794

[30] Friedberg MK , Fernandes FP , Roche SL , Grosse‐Wortmann L , Manlhiot C , Fackoury C , Slorach C , McCrindle BW , Mertens L , Kantor PF . Impaired right and left ventricular diastolic myocardial mechanics and filling in asymptomatic children and adolescents after repair of tetralogy of Fallot. Eur Heart J Cardiovasc Imaging. 2012;13:905–913. 22467442 10.1093/ehjci/jes067

[31] Koenigstein K , Raedle‐Hurst T , Hosse M , Hauser M , Abdul‐Khaliq H . Altered diastolic left atrial and ventricular performance in asymptomatic patients after repair of tetralogy of Fallot. Pediatr Cardiol. 2013;34:948–953. 23179427 10.1007/s00246-012-0584-1

[32] Lester SJ , Tajik AJ , Nishimura RA , Oh JK , Khandheria BK , Seward JB . Unlocking the mysteries of diastolic function: deciphering the Rosetta Stone 10 years later. J Am Coll Cardiol. 2008;51:679–689. 18279730 10.1016/j.jacc.2007.09.061

[33] Riesenkampff E , Mengelkamp L , Mueller M , Kropf S , Abdul‐Khaliq H , Sarikouch S , Beerbaum P , Hetzer R , Steendijk P , Berger F , Kuehne T . Integrated analysis of atrioventricular interactions in tetralogy of Fallot. Am J Physiol Heart Circ Physiol. 2010;299:H364–H371. 20495149 10.1152/ajpheart.00264.2010 PMC2930385

[34] Hardziyenka M , Campian ME , Verkerk AO , Surie S , van Ginneken AC , Hakim S , Linnenbank AC , de Bruin‐Bon HA , Beekman L , van der Plas MN , Remme CA , van Veen TA , Bresser P , de Bakker JM , Tan HL . Electrophysiologic remodeling of the left ventricle in pressure overload‐induced right ventricular failure. J Am Coll Cardiol. 2012;59:2193–2202. 22676940 10.1016/j.jacc.2012.01.063

[35] Hardziyenka M , Campian ME , Reesink HJ , Surie S , Bouma BJ , Groenink M , Klemens CA , Beekman L , Remme CA , Bresser P , Tan HL . Right ventricular failure following chronic pressure overload is associated with reduction in left ventricular mass: evidence for atrophic remodeling. J Am Coll Cardiol. 2011;57:921–928. 21329838 10.1016/j.jacc.2010.08.648

[36] Ponikowski P , Voors AA , Anker SD , Bueno H , Cleland JGF , Coats AJS , Falk V , Gonzalez‐Juanatey JR , Harjola VP , Jankowska EA , Jessup M , Linde C , Nihoyannopoulos P , Parissis JT , Pieske B , Riley JP , Rosano GMC , Ruilope LM , Ruschitzka F , Rutten FH , van der Meer P ; ESC Scientific Document Group . 2016 ESC guidelines for the diagnosis and treatment of acute and chronic heart failure: the task force for the diagnosis and treatment of acute and chronic heart failure of the European Society of Cardiology (ESC) developed with the special contribution of the Heart Failure Association (HFA) of the ESC. Eur Heart J. 2016;37:2129–2200. 27206819 10.1093/eurheartj/ehw128

[37] Yancy CW , Jessup M , Bozkurt B , Butler J , Casey DE Jr , Drazner MH , Fonarow GC , Geraci SA , Horwich T , Januzzi JL , Johnson MR , Kasper EK , Levy WC , Masoudi FA , McBride PE , McMurray JJ , Mitchell JE , Peterson PN , Riegel B , Sam F , Stevenson LW , Tang WH , Tsai EJ , Wilkoff BL . 2013 ACCF/AHA guideline for the management of heart failure: executive summary: a report of the American College of Cardiology Foundation/American Heart Association task force on practice guidelines. Circulation. 2013;128:1810–1852. 23741057 10.1161/CIR.0b013e31829e8807

[38] Geva T , Sandweiss BM , Gauvreau K , Lock JE , Powell AJ . Factors associated with impaired clinical status in long‐term survivors of tetralogy of Fallot repair evaluated by magnetic resonance imaging. J Am Coll Cardiol. 2004;43:1068–1074. 15028368 10.1016/j.jacc.2003.10.045

[39] Kempny A , Diller GP , Orwat S , Kaleschke G , Kerckhoff G , Bunck A , Maintz D , Baumgartner H . Right ventricular‐left ventricular interaction in adults with tetralogy of Fallot: a combined cardiac magnetic resonance and echocardiographic speckle tracking study. Int J Cardiol. 2012;154:259–264. 20937536 10.1016/j.ijcard.2010.09.031

[40] Movahed MR , Milne N . Poor correlation between left and right ventricular ejection fractions in patients with normal ventricular function. Exp Clin Cardiol. 2008;13:179–181. 19343163 PMC2663481

[41] Uebing A , Fischer G , Schlangen J , Apitz C , Steendijk P , Kramer HH . Can we use the end systolic volume index to monitor intrinsic right ventricular function after repair of tetralogy of Fallot? Int J Cardiol. 2011;147:52–57. 19716612 10.1016/j.ijcard.2009.07.031

[42] Bokma JP , de Wilde KC , Vliegen HW , van Dijk AP , van Melle JP , Meijboom FJ , Zwinderman AH , Groenink M , Mulder BJ , Bouma BJ . Value of cardiovascular magnetic resonance imaging in noninvasive risk stratification in tetralogy of Fallot. JAMA Cardiol. 2017;2:678–683. 28241248 10.1001/jamacardio.2016.5818 PMC5540000

